# Correlated Electronic Properties of a Graphene Nanoflake: Coronene

**DOI:** 10.3390/molecules24040730

**Published:** 2019-02-18

**Authors:** Suryoday Prodhan, Sumit Mazumdar, S. Ramasesha

**Affiliations:** 1 Solid State and Structural Chemistry Unit, Indian Institute of Science, Bangalore 560012, India; suryodayp@gmail.com; 2 Department of Physics, University of Arizona, Tucson, AZ 85721, USA; sumit@physics.arizona.edu; 3 College of Optical Sciences, University of Arizona, Tucson, AZ 85721, USA

**Keywords:** symmetrized DMRG, strongly-correlated system, carbon nanodots, Pariser–Parr–Pople (PPP) model, low-lying excited states

## Abstract

We report studies of the correlated excited states of coronene and substituted coronene within the Pariser–Parr–Pople (PPP) correlated π-electron model employing the symmetry-adapted density matrix renormalization group technique. These polynuclear aromatic hydrocarbons can be considered as graphene nanoflakes. We review their electronic structures utilizing a new symmetry adaptation scheme that exploits electron-hole symmetry, spin-inversion symmetry, and end-to-end interchange symmetry. The study of the electronic structures sheds light on the electron correlation effects in these finite-size graphene analogues, which diminishes going from one-dimensional to higher-dimensional systems, yet is significant within these finite graphene derivatives.

## 1. Introduction

In quantum chemical calculations of electronic structures of carbon (C)-based π-conjugated systems, the effects of electronic correlation have been probed by employing a number of techniques, most of which are variants of the restricted configuration interaction technique. Singles configuration interaction (SCI)-based techniques suffice for studying one-photon optical gaps in neutral carbon-based molecular systems, as these excitations are predominantly single electron-hole excitations [1]. However, for strongly-correlated low-dimensional systems, the shortcomings of the SCI are also well-documented [2,3,4,5,6,7,8,9,10]. Time-dependent density functional theory [11,12,13] or GWapproximation accompanied by Bethe–Salpeter correction [14,15,16,17] is essentially equivalent to the SCI approximation as only two-particle (one electron–one hole) interactions are included in these approaches and higher-order CIs are excluded.

While a full CI (FCI) study is the most preferred one, its use has been limited to ∼18-electron neutral systems, as the Hilbert space dimension increases exponentially with system size. Ab initio quantum chemical methods like CASPT2that are able to reproduce the energy spectra of correlated π-electron molecules correctly are limited to 8–10-electron neutral systems [18,19,20,21,22]. The multiple reference singles and double CI (MRSDCI) approach is another suitable iterative method for the introduction of higher-order CI effects, but the Hilbert space dimensions for different excited states, for similar accuracy, vary significantly [6,7,23,24]. On the other hand, the density matrix renormalization group (DMRG) method, introduced by White, is an accurate numerical many-body technique for studying low-lying states of one- and quasi-one-dimensional systems in real space [25,26,27,28,29]. In the DMRG method, similar to other renormalization group methods, the Hilbert space dimension remains fixed independent of the system sizes. For discrete molecular systems with energy gaps, the area law of entanglement entropy also holds, leading to high accuracy in the DMRG calculations [27,28], even with moderate dimensionality of the block state space.

The DMRG scheme, as usually implemented, utilizes conservation of the number of particles and the *z*-component of the total spin $S_{z}$. Thus, in this scheme, a few low-lying states are obtained in each of the sectors with a fixed number of particles and a fixed $S_{z}$ value. Although schemes that exploit other symmetries have been developed, they are limited to a few orbitals and a few particles. Yet, in studies of π-conjugated systems that probe the lowest-energy dipole-connected state, the lowest two-photon state, and the lowest triplet state, we need to exploit other symmetries like the electron-hole (*e*-*h*) symmetry, the spin-inversion symmetry, and the end-to-end interchange symmetry (see below). However, although application of the DMRG technique or its symmetrized versions is straightforward in one- and quasi-one-dimensional systems, it is not so trivial in higher dimensional systems like in graphene nanoflakes and graphene nanoribbons. The study of these large finite graphene analogues is expected to shed light on the properties in the thermodynamic limit and the effect of electronic correlation in these industrially important two-dimensional systems. Most of the earlier studies on graphene and graphene analogues have been done within a non-interacting model or employing the restricted configuration interaction technique with a few frontier molecular orbitals [30,31,32,33,34,35,36,37,38,39,40,41,42,43,44,45,46,47,48,49,50,51], while the importance of electron correlation in the electronic and magnetic structures of these systems has been emphasized in recent studies [23,24,52,53]. In the present paper, we demonstrate the application of the symmetrized DMRG technique in the study of a graphene nanoflake, coronene, within a long-range correlated π-electron model. This molecule has recently been studied employing the MRSDCI approach [23,24,54], and we reexamine the earlier results. We also study the effect of weak donor-acceptor substitutions, which lower the symmetry of the overall molecule. Transition dipole moments to the low-lying optical states along with two-photon absorption cross-sections for the low-lying two-photon states are also calculated.

The paper is organized as follows. In the next section, we give an account of the model Hamiltonian employed in our study along with a brief discussion about the DMRG technique and the symmetries utilized in our calculations. In Section 3, we present our results for coronene and substituted coronene. In the last section, we present our conclusions.

## 2. Methodology

Ab initio DMRG calculations for neutral systems employing molecular orbitals have bottlenecks since the calculation of two-electron integrals is computationally expensive, and consequently, these studies employ ∼50 active space orbitals [55]. On the other hand, DMRG calculations with localized orbitals have been successfully employed for π-conjugated systems with several hundred $p_{z}$ orbitals within the PPP Hamiltonian [53,56,57,58,59]. The ab initio study of arenes using the DMRG method has also revealed that fully-localized orbitals bring about faster convergence of energies compared to canonical Hartree–Fock orbitals or split-localized orbitals [55], making the localized description the picture of choice.

### 2.1. Model Hamiltonian

We consider the PPP π-electron Hamiltonian [60,61], which is a widely-employed semi-empirical model for studying the behavior of C-based π-conjugated systems [3,4,5,6,7,8,9]. The PPP Hamiltonian is given by,

(1) $$\begin{array}{cc} \hat{H} & =\sum_{<i,j>,\sigma }t_{0}\left({\hat{c}}_{i,\sigma }^{†}{\hat{c}}_{j,\sigma }^{}+H.C.\right)+\sum_{i}\epsilon_{i}{\hat{n}}_{i}\\ & +\sum_{i}\frac{U}{2}{\hat{n}}_{i}\left({\hat{n}}_{i}-1\right)+\sum_{i>j}V_{ij}\left({\hat{n}}_{i}-z_{i}\right)\left({\hat{n}}_{j}-z_{j}\right) \end{array}$$

In the above, ${\hat{c}}_{i,\sigma }^{†}$ (${\hat{c}}_{i,\sigma }^{}$) creates (annihilates) a π-electron with spin σ on the $p_{z}$ orbital on C-atom *i*; ${\hat{c}}_{i,\sigma }^{†}{\hat{c}}_{i,\sigma }^{}$ is the corresponding number operator, and $n_{i}=\sum_{\sigma }c_{i,\sigma }^{†}{\hat{c}}_{i,\sigma }$ is the total number operator. Here, $t_{0}$ is the transfer integral between bonded C-atoms *i* and *j*, $\epsilon_{i}$ is the site energy of the *i*th C-atom, *U* is the repulsive Hubbard interaction between two electrons occupying the same $p_{z}$ orbital, and $V_{ij}$ is the long-range electron correlation between C-atoms *i* and *j*. The latter is obtained from the Ohno interpolation scheme [62,63] (Equation (2)),

(2) $$V_{ij}=14.397{\left[{\left(\frac{14.397}{U}\right)}^{2}+r_{ij}^{2}\right]}^{-\frac{1}{2}}$$

where the distance $r_{ij}$ between the C-atoms iand j are in Å units, while the Hubbard interaction energy term *U* is in electron-volts (eVs). Finally, $z_{i}$ is the local chemical potential, expressed by the number of π-electrons on C-atom *i*, which leaves the site neutral ($z_{i}=1$ for C-atoms). In our calculations, we have used standard PPP parameters, $t_{0}=-2.40$ eV and U=11.26 eV, which have been widely used over the past several decades [64,65]; the employed on-site correlation energy *U* is the sum of the ionization potential and the electron affinity of C in a $sp^{2}$-hybridized system [64]. Substitution effects can be probed by introducing positive or negative site energies $\epsilon_{i}$, to mimic donor and acceptor groups, respectively, while the site energy for unsubstituted C-atoms is taken as zero.

### 2.2. The DMRG Technique

In the DMRG technique, the full system is divided into two blocks, generally referred to as the left (L) and right (R) blocks, which are iteratively grown by a few sites (usually one) at each step. The complete wavefunction of the system is represented in the direct product space of the block states. The block space of each individual block is approximated by retaining reduced density matrix eigenvectors of the corresponding block with highest eigenvalues, and the exponentially-growing Hilbert space of a many-body system becomes well adapted to a basis space of fixed dimension, independent of the system size. The reduced density matrix of a particular block is obtained by employing full system eigenstates while assuming the other block as the environment, followed by its diagonalization to obtain the block states. Matrices of the block Hamiltonians and of the individual site operators are constructed at the *l*th step in the direct product basis of old block states (obtained in the step l−1) and Fock states of the newly-added sites. Afterwards, these matrices are renormalized employing the new block states constructed at the *l*th step. In the next step, the system is grown by adding a few sites to both the left and right blocks, and the full system Hamiltonian is constructed in the direct product basis of the block states of the left and right blocks along with the Fock states of the newly-added sites. The full Hamiltonian matrix is diagonalized to obtain targeted eigenstates of the system, which are then used to study different physical properties. The above procedure, known as the infinite DMRG method, is iteratively repeated until the desired system size is reached.

Although the infinite DMRG algorithm can be employed to study physical properties at the polymeric limit, for finite-size systems, the accuracy of the calculation can be significantly improved by the finite DMRG algorithm. In the infinite scheme, the block states at the intermediate steps do not correspond to the final system. This flaw can be resolved through construction of block spaces employing wavefunctions corresponding to the final system size. The procedure is termed as “sweeping”, where iteratively, one block is grown at the expense of the other block, while keeping the final system size fixed. At the final step of a full-sweep, the sizes of the two blocks become N/2−1 where *N* is the final system size. The finite DMRG procedure is a non-trivial, but essential procedure for the study of molecular systems as the energies improve significantly following the sweeping procedure.

For quasi-one-dimensional systems, the order in which the new sites are added to build the molecule is important to attain high accuracy. For the same DMRG cut-off, different sequences of adding new sites give different energy eigenvalues. This is well known in the ab initio DMRG approaches where the order of adding orbitals is determined by the entanglement [66,67,68]. The order in which the sites are added to build the molecule coronene and its derivative is as follows. At every stage of the infinite DMRG iteration, transfer terms are introduced between the sites added earlier (old sites) and the new sites, as well as between old sites in the two blocks. We add the sites in such a way that the interaction between the old site and the new site at any given stage involves as recent an old site as possible. This implies that the interaction between the new site and the old site is such that the old site operators have been renormalized the fewest number of times possible. In the early DMRG studies of systems with periodic boundary condition, this particular requirement was restored by placing one of the newly-added sites between the old blocks while placing the other new site at the end of a particular old block (L or R).

**Figure 1:**
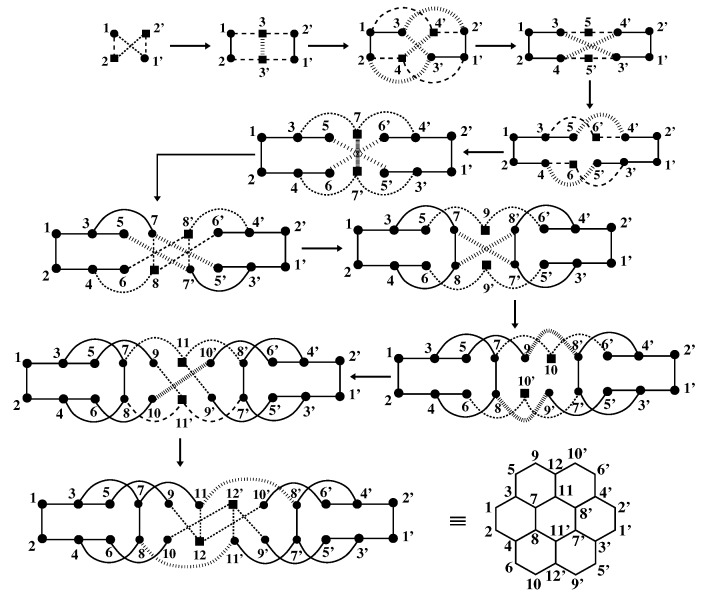
Construction of the coronene molecule in the infinite DMRG method starting from a small system (four sites). The number of connections between the new and the old sites at the intermediate steps are kept similar to that in the final system for higher accuracy. At every step of the algorithm, two new sites are added, one to the system block (L) and the other to the environment block (R). The sites in the *L*-block are denoted by unprimed numbers, while those in the *R*-block are denoted by primed numbers. The newly-added sites are denoted by filled squares (*▪*), while old sites are denoted by filled circles (•). Solid lines are bonds within a block. The broken lines denote the connections between • and *▪*. Bonds between the two blocks, as well as the bond between newly-added sites are denoted by hatched lines.

Figure 1 shows the steps of building the coronene molecule starting from a four-atom ring and adding two new atoms at each step. The noninteracting Hückel model for this molecule ($\epsilon_{i}\;=\;U\;=\;V_{ij}\;=\;0$ in Equation (1)) can be solved trivially for ground and low-lying excited states. The same can also be obtained using the DMRG algorithm. The comparison of the two results (see Table 1) provides a stringent check on the DMRG accuracy, since within the noninteracting model, the system block and the environment block are more entangled, as compared to interacting models with site-diagonal interactions, such as the Hubbard and the PPP models [69]. We find that for coronene, the ground state energy is accurate to 0.17% and the optically-excited state is accurate to 0.38%, while the optical gap (energy difference between the two) is accurate to 6.1% (the exact gap is 1.07838 $t_{0}$). Since the accuracy of the DMRG method increases with decreasing entanglement, the Hückel model provides an upper limit for the errors in the correlated models. Additionally, the comparison of the DMRG calculations against the Hückel model can be employed as an effective tool to calibrate the required block space dimension for desired numerical accuracy within these finite-size quasi-one-dimensional systems.

### 2.3. Symmetries of the Hamiltonian

We are particularly interested in the low-energy one- and two-photon excited states along with the low-lying triplet states of the molecules. However, the number of energy states that reside between the ground state and the desired states can be variable and large. Hence, targeting “important” states is an almost impossible task without invoking the basic symmetries of the full Hamiltonian. In the present study, we have utilized the end-to-end interchange symmetry $\left(C_{2}\right)$, *e*-*h* symmetry, and spin-flip or parity symmetry (P) of the full system Hamiltonian within the DMRG framework employing a modified algorithm for symmetry adaptation [70]. In this algorithm, the symmetry operators are expressed as extremely sparse matrices, with only one non-zero element per row and column. Consequently, we get rid of the computationally-expensive Gram–Schmidt orthonormalization procedure during the construction of the symmetry-adapted basis states.

The PPP Hamiltonian conserves total spin (S), but it is difficult to adopt total spin conservation within the DMRG scheme. In order to target states with different *S*, we exploit the spin-flip symmetry (P) in the $S_{z}=0$ sector, where the Hamiltonian remains invariant as all spins of the system are reversed. The symmetry bifurcates the $S_{z}=0$ space into one subspace with even total spin (designated as “e”) and another with an odd total spin (designated as “o”). In addition, the Hamiltonian for a neutral bipartite system remains invariant under *e*-*h* symmetry, where the creation (annihilation) operator of one sub-lattice is interchanged by the annihilation (creation) operator, while in the other sub-lattice, the interchange accompanies a phase factor of −1. The eigenstates can be labeled “+” or “−” depending on the eigenvalue (+1 or −1) while operated by the *e*-*h* symmetry operator. Finally, the full system eigenstates can be labeled *A* or *B*, based on their parity (even or odd) with respect to the $C_{2}$ operation.

The three symmetry operators and their products along with identity form an Abelian group, which sub-divides the $S_{z}=0$ space into eight subspaces. In general, the ground state has an even character with respect to every symmetry operation and lies in the ${}^{e}A^{+}$ subspace. Optical one-photon states remain in the ${}^{e}B^{-}$ space, while the two-photon states have the same symmetry characteristics as the ground state. The lowest triplet state energy is in the $S_{z}=1$ space where the *P* symmetry cannot be employed, and it remains in the $B^{+}$ space.

In each symmetry subspace, we have calculated a few low-lying eigenstates of the Hamiltonian to ascertain the spectra in the low energy region. However, for the calculation of the transition dipole moments, the average reduced density matrix is employed instead of the reduced density matrix corresponding to a single state, in order to attain a common block space description. The average reduced density matrix [26] is defined by $\rho =\sum_{i}\omega_{i}\rho_{i}$ where $\rho_{i}$ is the reduced density matrix corresponding to eigenstates |i〉. $\omega_{i}$ are the weights of the corresponding eigenstates, which we have taken as $\omega_{i}=1/W$, where *W* is the number of low-lying eigenstates computed in the symmetry subspace. The block states obtained from the average reduced density matrix are employed for the DMRG calculations. The magnitude of the cut-off in the block space dimension does not lead to admixture of the different symmetry states in the state-average DMRG calculations, since we have retained all symmetry partners of the block states in our algorithm.

### 2.4. Two-Photon Absorption Cross-Section

Two-photon absorption (TPA) is a third-order nonlinear optical process, which involves simultaneous absorption of two photons and is related to the imaginary part of the second-order hyperpolarizability $\chi^{\left(3\right)}\left(-\omega ;\omega ,-\omega ,\omega \right)$, where ℏω is half of the excitation energy of the two-photon state |TP〉$\left(\hbar \omega =\left(E_{TP}-E_{G}\right)/2\right)$. We have employed the correction vector (CV) technique to compute the TPA cross-section. The first-order CV is calculated employing the inhomogeneous linear algebraic equation,

(3) $$\left(\hat{H}-E_{G}+\hbar \omega \right)|\phi_{i}^{\left(1\right)}\left(\omega \right)\rangle ={\tilde{\mu }}_{i}|G\rangle$$

where *H* is the Hamiltonian matrix, |G〉 is the ground state with energy $E_{G}$, and ${\tilde{\mu }}_{i}$ is the *i*th component of the dipole displacement operator, ${\tilde{\mu }}_{i}={\hat{\mu }}_{i}-\langle G|{\hat{\mu }}_{i}|G\rangle$ (i=x,y,z). The linear algebraic equations are solved efficiently employing the small matrix algorithm developed by Ramasesha [71]. Upon expansion in the basis of the excited states {|R〉}, the correction vector can be written as,

(4) $$|\phi_{i}^{\left(1\right)}\left(\omega \right)\rangle =\sum_{R}\frac{\langle R|{\tilde{\mu }}_{i}|G\rangle }{E_{R}-E_{G}+\hbar \omega }|R\rangle$$

The expression for the ijth element of the two-photon transition matrix is given by [72],

(5) $$\begin{array}{cc} S_{ij}\left(\omega \right) & =\sum_{R}\left[\frac{\langle G|{\tilde{\mu }}_{i}\left|R\rangle \langle R\right|{\tilde{\mu }}_{j}|TP\rangle }{E_{R}-E_{G}-\hbar \omega }+\frac{\langle G|{\tilde{\mu }}_{j}\left|R\rangle \langle R\right|{\tilde{\mu }}_{i}|TP\rangle }{E_{R}-E_{G}-\hbar \omega }\right]\\ & =\langle \phi_{i}^{\left(1\right)}\left(-\omega \right)|{\tilde{\mu }}_{i}|TP\rangle +\langle \phi_{j}^{\left(1\right)}\left(-\omega \right)|{\tilde{\mu }}_{i}|TP\rangle \end{array}$$

while the TPA cross-section for a linearly-polarized monochromatic light of a randomly-oriented sample as in the solution or gas phase is given by [72],

(6) $$\delta_{TPA}=\frac{1}{15}\sum_{i,j=x,y,z}\left(S_{ii}S_{jj}^{*}+2S_{ij}S_{ij}^{*}\right)$$

## 3. Computational Results

**Figure 2:**
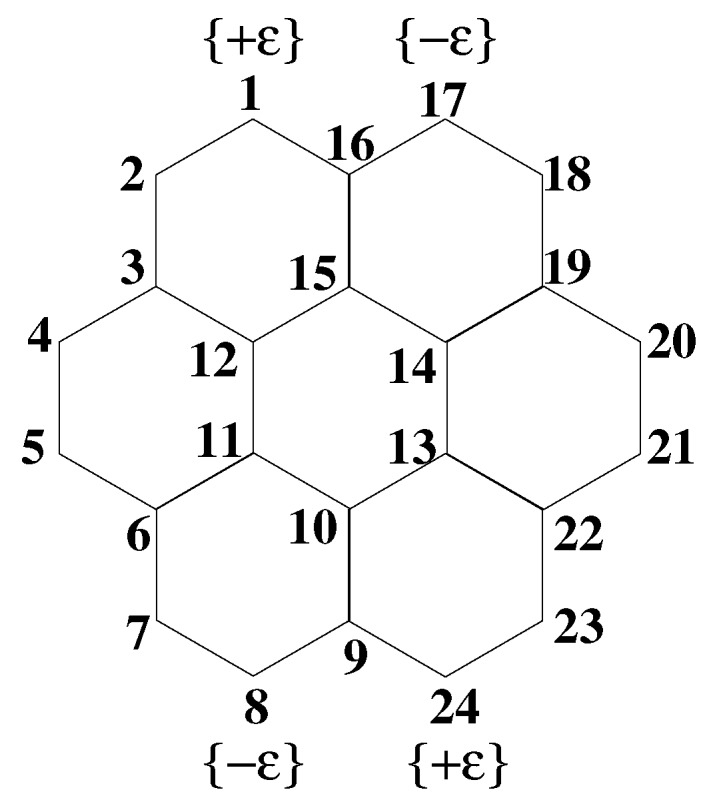
(Color online) Schematic diagram of the coronene molecule. The sites of substitution in substituted coronene are also indicated; +ϵ represents a donor site, while −ϵ represents an acceptor site.

We have studied a few low-lying states of coronene in different symmetry subspaces within the PPP model, and the relevant energy gaps, defined as differences from the ground state, are tabulated in Table 2. We have also calculated energies in the corresponding symmetry subspaces for the substituted coronene of Figure 2 (see Table 3), with donor and acceptor groups of equal strength (|ϵ|=1.0 eV). For all our calculations, we maintained a truncated block space dimension of ∼1000. We have used a single spatial symmetry, the $C_{2}$ symmetry whose axis is perpendicular to the molecular plane, as our DMRG calculation cannot handle more than one symmetry axis. We labeled all the eigenstates using the $D_{2h}$ subgroup symmetry, which is simpler than determining the irreducible representations of the $D_{6h}$ point group symmetry. In the $D_{6h}$ point group representation, allowed optical transitions from the ground state with $A_{1g}$ symmetry are only to doubly-degenerate $E_{1u}$ states, whose transition dipoles lie in the molecular plane. In the $D_{2h}$ subgroup, these states remain as two-fold degenerate $B_{2u}$ and $B_{3u}$ states, respectively, but now, their transition dipoles lie strictly along orthogonal *y*- and *x*-axes, respectively. Consequently, use of the $D_{2h}$ symmetry subgroup instead of $D_{6h}$ simply implies that the doubly-degenerate optical states, with mixed polarizations in the molecular plane, are assumed to have distinct polarizations along the Cartesian axes and *no information has been lost*. Within the DMRG calculations of these doubly-degenerate states, however, calculated transition dipoles along any one direction pick up a weak orthogonal component even when $D_{2h}$ symmetry is employed. The lowest optical states obtained may not be the states with the highest transition dipole moment, which will be prominent in UV-visible spectroscopy, but corresponds to the lowest energy state of the appropriate symmetry subspace [23].

### 3.1. Correlation Strength and Ordering of Excited States

As has been shown explicitly in calculations of linear polyenes [2,73,74,75], the most important consequence of strong electron correlations is the energy ordering of excited states according to their ionicities. In the language of valence bond (VB) theory, eigenstates are covalent if they are dominated by VB diagrams in which the $p_{z}$ orbitals of C-atoms are neutral, i.e., singly occupied. Similarly, eigenstates are ionic if in the dominant VB diagrams, there occurs at least one pair of $p_{z}$ orbitals that are positively and negatively charged. One simple measure of the ionicity within the PPP Hamiltonian of Equation (1) is the expectation value of $\langle n_{i,↑}n_{i,↓}\rangle$, which measures the probability of double occupancy in the $p_{z}$ orbital at site *i*. Within the noninteracting Hückel model, $\langle n_{i,↑}n_{i,↓}\rangle$ is exactly 0.25 for the ground state. The ground state of the interacting Hamiltonian is more covalent than that of the noninteracting Hamiltonian, and $\langle n_{i,↑}n_{i,↓}\rangle <0.25$ for the former. Experimentally, the ionicities of the excited states are a more relevant quantity. Within the noninteracting model, there is no one-to-one correspondence between energies of states and their ionicities. Within the interacting model, however, predominantly covalent states occur energetically below predominantly ionic states.

Fortunately, this relative ordering, lowest covalent excited states occurring below lowest ionic states, can be tested optically in centrosymmetric systems with distinct one-photon and two-photon states. As was recognized long ago, dipole selection rules in centrosymmetric systems dictate that transition dipole matrix elements are nonzero only between states with opposite parity and *e*-*h* symmetries [2,9]. In the context of neutral π-conjugated molecules, this means that the one-photon transition from the even parity $eA^{+}$ covalent ground state can occur only for odd parity $eB^{-}$ states. Note that this also implies that the lowest two-photon states, which are dipole-coupled to the $eB^{-}$ states, are covalent (see Equation (5)) and are hence likely to occur below the lowest one-photon state. The occurrence of the lowest two-photon state below the lowest one-photon state has been experimentally confirmed in linear polyenes [73].

The theoretical measure of the correlation strength for a charge-neutral C-based molecule to a first approximation is the ratio of the effective on-site correlation to the width of the one-electron energy spectrum. The effective on-site correlation within the PPP model is $U_{PPP}∼\left(U-V_{12}\right)$, where $V_{12}$ is the nearest-neighbor Coulomb interaction. This quantity is independent of dimensionality. In contrast, the width of the one-electron energy spectrum increases with dimensionality, implying therefore that the effective correlation strength is smaller in two dimensions than in one dimension. The relative energies of the lowest one- versus two-photon states in graphene nanofragments can therefore not be guessed based on the known results for polyenes.

Aside from its covalent nature, another aspect of the lowest two-photon state in linear polyenes has been of interest, viz. its characterization as a bound state of the two lowest triplet excitations T${}_{1}$ of the polyene [6,75]. Indeed, several of the lowest two-photon states in linear polyenes are superpositions of triplet excitations T${}_{1}$, as well as higher energy T${}_{2}$, T${}_{3}$, etc. (the two triplets that constitute an excited covalent singlet need not be identical) [6]. In other words, even parity covalent excited states in linear polyenes are necessarily superpositions of two triplets. It is not a priori clear that this will hold true in polycyclic aromatic hydrocarbons such as coronene, where there occur C-atoms that are different, peripheral versus internal, as well as bi-coordinate versus tri-coordinate.

In Table 2, we have listed the energies of the lowest optical states, the lowest triplet states, and the lowest two-photon states, relative to the ground state. We have also included the lowest states that are optically dark under both one- and two-photon excitation. The latter are equivalent to the covalent $B_{u}^{+}$ states of polyenes [75], each of which can be viewed as a superposition of covalent $B_{u}$ triplets $T_{i}$ and $T_{j}$, i≠j. Because of our use of a single C${}_{2}$ symmetry element, we are unable to distinguish between $A_{g}^{+}$ and $B_{1g}^{+}$ states that are degenerate in the noninteracting limit, but are nondegenerate within the correlated PPP Hamiltonian [23]. The ${}^{1}$B${}_{2u}^{-}$ and ${}^{1}$B${}_{3u}^{-}$ states are degenerate, but the triplet states ${}^{3}B_{2u}^{+}$ and ${}^{3}B_{3u}^{+}$ need not be degenerate in coronene [23]. We have therefore assigned the triplet at 2.35 eV to $1^{3}B_{2u}^{+}$/$1^{3}B_{3u}^{+}$, while the one at 3.0 eV is assigned to $1^{3}B_{3u}^{+}$/$1^{3}B_{2u}^{+}$. We have given similar labels to the lowest two-photon states, where two multiple assignments are possible.

The lowest two-photon energy along with the lowest triplet energy calculated in coronene are in excellent agreement with the previously-reported values by Aryanpour et al., obtained using the MRSDCI technique [23]. The calculated energy of the lowest two-photon state (that occurs in the ${}^{1}A_{g}^{+}$ subspace) reported earlier was 3.96 eV for coronene, to be compared against 3.97 eV found in our calculations. These numbers match very well against the experimental two-photon absorption spectrum [23]. The earlier reported lowest triplet energy was 2.38 eV (the experimentally-identified peak was at ∼2.40 eV, probed by phosphorescence and electron energy loss spectroscopy [81,83]), which was also close to the 2.35 eV obtained by us. However, the lowest one-photon energy and relative position of the lowest optical state with respect to the lowest two-photon state do not agree well with the earlier study [23,24]. In coronene, the energy of the lowest optical $1\;{}^{1}B_{2u}^{-}$ and $1\;{}^{1}B_{3u}^{-}$ states calculated earlier [23,24] was 4.11 eV, while we found two nearly-degenerate excitations at higher energies of 4.83 and 4.87 eV, respectively.

The experimental linear absorption spectrum of coronene shows a prominent absorption band in this region with the maximum at 4.06–4.30 eV, both in the solution [23,76,77,78,79,80,85] and vapor phase [81,82,83,84]. The discrepancy between our result and the previous computational result arises from the use of “bare” PPP-Ohno parameters in the present work as opposed to “screened” parameters in the previous work [23,24].

We see in Table 2 a low-lying state at an energy of 3.88 eV, with a small transition dipole moment. This state was found to be optically forbidden in the MRSDCI calculations [23,24]. The weak dipole coupling found in the present calculations probably results from our incorporation of only one $C_{2}$ symmetry axis or our use of the average density matrices, obtained from eigenstates of different symmetry subspaces, which can lead to weak spatial symmetry violation. Indeed, we revisited the calculations without employing the $C_{2}$ symmetry and found that the energy of this state remains unchanged. However, this state lies in the “−” subspace of *e*-*h* symmetry with a small transition dipole moment. Therefore, we conclude that this state corresponds to the $1\;{}^{1}A_{g}^{-}/1\;{}^{1}B_{1g}^{-}$ state, in agreement with the previous study [23,24]. The calculated small transition dipole moment is thus an artifact, although weak violation of *e*-*h* symmetry, as would occur in the real molecule, can lead to observable absorption. Indeed, as pointed out in the earlier theory-experiment work [23], this “forbidden” state is seen as a weak absorption experimentally.

The 2.82-eV excitation in coronene, on the other hand, is strictly forbidden, as it belongs to the same *e*-*h* symmetry subspace as the ground state. This argument is supported by the fact that this state acquires some intensity on breaking the *e*-*h* symmetry by introducing substituents, as can be seen from Table 3. The appearance of the absorption peak at ∼2.95 eV in thin films of coronene also suggests the presence of a singlet state close to the lowest triplet state [86,87].

Substitution by donor-acceptor groups does not seem to have an appreciable effect on the energy gaps between the states, although the lifting of *e-h* symmetry allows optical transitions to states that are dipole forbidden in the unsubstituted molecule. The extent of mixing of different symmetry states of the unsubstituted system due to substitution depends on the strength of the donor-acceptor groups.

The spin gap (energy difference between lowest triplet state and ground state) is also a good measure of the effective correlation strength. The stronger the effective correlation, the smaller is the spin gap. Based on the similar spin gaps in the unsubstituted versus substituted molecules, we conclude that the effective correlation strengths are the same in the two molecules. This indicates that the previous claim of a stronger correlation effect in lower symmetry molecules [24] may be an oversimplification.

**Figure 3:**
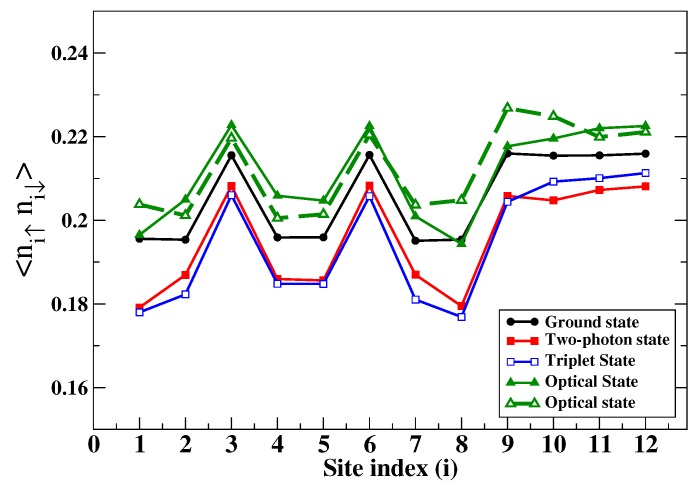
(Color online) The probability of double occupancy of C-atoms by electrons plotted against the site index (see Figure 2) for coronene. Sites related by $C_{2}$ symmetry are perfectly equivalent, and hence, are not shown. Lines are guides to the eye only. The two different plots for the optical states correspond to the two nearly degenerate states.

In Figure 3, we have plotted the $\langle n_{i,↑}n_{i,↓}\rangle$ for each of the C-atoms in coronene, for the ground state, the optical $1\;{}^{1}B_{2u}^{-}$ and $1\;{}^{1}B_{3u}^{-}$ states, the lowest two-photon state at 3.97 eV, and the lowest triplet state. As expected, $\langle n_{i,↑}n_{i,↓}\rangle$ for the ground state was smaller than 0.25 for all C-atoms, indicating its covalent character. The same expectation value was larger for the optical state, also as anticipated for this ionic state. Interestingly, $\langle n_{i,↑}n_{i,↓}\rangle$ for the lowest triplet and the lowest two-photon state were both smaller than that of the ground state, indicating (i) a covalent characteristic larger than that of the ground state and (ii) a nearly equal covalent characteristic in both. The equality between the lowest triplet and the lowest two-photon state is surprising, given that the latter is not a simple two-triplet state.

### 3.2. TPA Cross-Section

In Table 4, we have tabulated the TPA cross-sections for low-lying two-photon states in coronene along with two-photon transition matrix elements. In coronene, we found that the higher energy two-photon state $3\;{}^{1}A_{g}^{+}$/$2\;{}^{1}B_{1g}^{+}$ had a larger TPA cross-section than that of the lowest two-photon states. This theoretical result is in qualitative agreement with the experimental solution two-photon measurements in this energy region. Since the two Cartesian axes are equivalent in coronene, the transition matrix elements $S_{xx}$ and $S_{yy}$ should be nearly the same. However, in our calculations, we found $|S_{yy}\left|>\right|S_{xx}|$ in most of the cases, which we attribute to the fact that we have used only one $C_{2}$ symmetry axis while targeting the states, as well as to the use of average density matrices. When the states are nearly degenerate, these approximations could break the true symmetry, which the eigenstates will otherwise possess.

## 4. Discussion and Conclusions

We have studied the lowest energy states and their relative orderings in two finite centrosymmetric graphene nanoflakes within the PPP π-electron Hamiltonian, using the DMRG approach. Electron correlations drive covalency in both molecules and also change the relative orderings of one- versus two-photon excitations. As in linear polyenes, the lowest triplet and the lowest two-photon states are covalent in the language of VB theory and occur below the lowest one-photon optical state. The proximity in energy between the ionic one-photon state and the covalent two-photon state, relative to that in the polyenes, however, is an indication of a relatively weaker correlation effect in these two-dimensional molecules with a wider one-electron energy spectrum. Additionally, the lowest two-photon state is not a simple two-triplet state, unlike in the polyenes. We believe that this is a consequence of the different topology in these polycyclic hydrocarbons, in which there occur C-atoms with both two and three nearest neighbors. The occurrence of higher energy two-photon states that are two-triplets [24] indicates that in these two-dimensional molecules, there occur two different kinds of covalent states, which may or may not be simply classified as two-triplets. This relationship between the nature of covalent states with topology, along with the correlation effects in graphene fragments of a larger and larger size are topics of ongoing and future interest. 

References

## Figures and Tables

**Table 1:** Ground and lowest optical state energies of coronene within the non-interacting Hückel model, in units of $t_{0}$, calculated using the Hückel and symmetrized DMRG approaches.

Nature of the State	Hückel	Symmetrized DMRG
Ground state	−34.57183	−34.51000
Optical state	−33.49345	−33.36570

**Table 2:** Energies of low-lying two-photon states, optical states, triplet states, and a few other optically-dark states in coronene, relative to the ground state. Although coronene has $D_{6h}$ symmetry, here, the states are labeled by the symmetry representations of its subgroup $D_{2h}$. Whenever a state cannot be uniquely labeled due to lower symmetry employed in the study, both possible labels for the state have been given. The transition dipole moment (in Debye) from the ground state to the excited states $\left(\mu_{tr,x/y}\right)$ along specific axes is also specified in the last two columns. Energies determined by UV-visible spectroscopy are also mentioned in Footnotes (a) and (c).

Coronene				
**Nature of the State**	**State**	**Energy Gap (eV)**	$\mu_{\mathrm{tr},x}\left(D\right)$	$\mu_{\mathrm{tr},y}\left(D\right)$
Two-photon	$\left(2\;{}^{1}A_{g}^{+}/1\;{}^{1}B_{1g}^{+}\right)$	3.97	0.00	0.00
	$\left(1\;{}^{1}B_{1g}^{+}/2\;{}^{1}A_{g}^{+}\right)$	4.09	0.00	0.00
	$\left(3\;{}^{1}A_{g}^{+}/2\;{}^{1}B_{1g}^{+}\right)$	5.08	0.00	0.00
Optical	$1\;{}^{1}B_{2u}^{-}$	4.83	0.81	8.43
	$1\;{}^{1}B_{3u}^{-}$	4.87	7.51	0.49
Triplet	$\left(1\;{}^{3}B_{2u}^{+}/1\;{}^{3}B_{3u}^{+}\right)$	2.35	0.00	0.00
	$\left(1\;{}^{3}B_{3u}^{+}/1\;{}^{3}B_{2u}^{+}\right)$	3.00	0.00	0.00
	$\left(2\;{}^{3}B_{2u}^{+}/2\;{}^{3}B_{3u}^{+}\right)$	3.02	0.00	0.00
	$\left(1\;{}^{3}A_{g}^{+}/1\;{}^{3}B_{1g}^{+}\right)$	3.35	0.00	0.00
Dark states	$\left(1\;{}^{1}B_{2u}^{+}/1\;{}^{1}B_{3u}^{+}\right)$	2.82	0.00	0.00
	$\left(1\;{}^{1}A_{g}^{-}/1\;{}^{1}B_{1g}^{-}\right)$	3.88	0.28	1.74
	$\left(1\;{}^{1}B_{1g}^{-}/1\;{}^{1}A_{g}^{-}\right)$	4.73	0.00	0.00

${}^{1}$4.10 eV [23]; 4.06–4.27 eV [76]; 4.21 eV [77]; 4.07–4.23 eV [78]; 4.12–4.44 eV [79]; 4.06–4.27 eV [80]; 4.28 eV [81]; 4.06 eV [82]; 4.09 eV [83]; 4.27 eV [84]. ${}^{2}$ Non-zero value of transition dipole moment along the polarization direction forbidden by symmetry is an artifact of the calculations as average density matrices, calculated from eigenstates of different symmetry subspaces, are employed to determine the transition dipole moment. However, the errors are negligible as intensities depend on the square of the transition dipole moment. ${}^{3}$ 2.40 eV [81,83].

**Table 3:** Energies of the low-lying states states in substituted coronene are tabulated below. The transition dipole moment (in Debye) from the ground state to the excited states $\left(\mu_{tr,x/y}\right)$ along specific axes is also specified in the last two columns.

Substituted Coronene				
**Nature of the State**	**State**	**Energy Gap (eV)**	$\mu_{\mathrm{tr},x}\left(D\right)$	$\mu_{\mathrm{tr},y}\left(D\right)$
Two-photon	$2\;{}^{1}A_{g}$	4.01	0.00	0.00
	$3\;{}^{1}A_{g}$	4.81	0.00	0.00
	$4\;{}^{1}A_{g}$	4.90	0.00	0.00
Optical	$1\;{}^{1}B_{u}$	2.90	0.00	0.23
	$2\;{}^{1}B_{u}$	3.87	0.12	1.46
	$3\;{}^{1}B_{u}$	5.18	0.17	7.52
	$4\;{}^{1}B_{u}$	5.97	4.10	0.59
Triplet	$1\;{}^{3}B_{u}$	2.29	0.00	0.00
	$2\;{}^{3}B_{u}$	2.92	0.00	0.00
	$3\;{}^{3}B_{u}$	3.48	0.00	0.00

**Table 4:** Two-photon transition matrix elements along with the two-photon absorption (TPA) cross-section for the lowest two-photon states in the unsubstituted coronene molecule. Possible symmetry labels are provided wherever a unique symmetry label cannot be determined. Transition matrix elements, as well as TPA cross-sections are given in atomic units.

	Two-Photon State	$S_{\mathrm{xx}}$	$S_{\mathrm{yy}}$	$S_{\mathrm{xy}}$	$\delta_{\mathrm{TPA}}$
	$\left(2\;{}^{1}A_{g}^{+}/1\;{}^{1}B_{1g}^{+}\right)$	10.68	−67.05	−0.97	821.56
Coronene	$\left(1\;{}^{1}B_{1g}^{+}/2\;{}^{1}A_{g}^{+}\right)$	1.94	−0.97	−35.95	349.39
	$\left(3\;{}^{1}A_{g}^{+}/2\;{}^{1}B_{1g}^{+}\right)$	23.32	−69.96	1.94	868.77
